# Survival Analyses for Patients With Surgically Resected Pancreatic Neuroendocrine Tumors by World Health Organization 2010 Grading Classifications and American Joint Committee on Cancer 2010 Staging Systems

**DOI:** 10.1097/MD.0000000000002156

**Published:** 2015-12-07

**Authors:** Min Yang, Neng-wen Ke, Lin Zeng, Yi Zhang, Chun-lu Tan, Hao Zhang, Gang Mai, Bo-le Tian, Xu-bao Liu

**Affiliations:** From the Department of Pancreatic Surgery (MY, N-wK, YZ, C-lT, HZ, GM, B-lT, X-bL); General Ward of Sports Medicine & Cardiopulmonary Rehabilitation (LZ), West China Hospital of Sichuan University, Chengdu, Sichuan Province, People's Republic of China.

## Abstract

In 2010, World Health Organization (WHO) reclassified pancreatic neuroendocrine tumors (p-NETs) into 4 main groups: neuroendocrine tumor G1 (NET G1), neuroendocrine tumor G2 (NET G2), neuroendocrine carcinoma G3 (NEC G3), mixed adeno and neuroendocrine carcinoma (MANEC). Clinical value of these newly updated WHO grading criteria has not been rigorously validated. The authors aimed to evaluate the clinical consistency of the new 2010 grading classifications by WHO and the 2010 tumor-node metastasis staging systems by American Joint Committee on Cancer (AJCC) on survivals for patients with surgically resected p-NETs. Moreover, the authors would validate the prognostic value of both criteria for p-NETs.

The authors retrospectively collected the clinicopathologic data of 120 eligible patients who were all surgically treated and histopathologically diagnosed as p-NETs from January 2004 to February 2014 in our single institution.

The new WHO criteria were assigned to 4 stratified groups with a respective distribution of 62, 35, 17, and 6 patients. Patients with NET G1 or NET G2 obtained a statistically better survival compared with those with NEC G3 or MANEC (*P* < 0.001). Survivals of NET G1 was also better than those of NET G2 (*P* = 0.023), whereas difference of survivals between NEC G3 and MANEC present no obvious significance (*P* = 0.071). The AJCC 2010 staging systems were respectively defined in 61, 36, 12, and 11 patients for each stage. Differences of survivals of stage I with stage III and IV were significant (*P* < 0.001), as well as those of stage II with III and IV (*P* < 0.001); whereas comparisons of stage I with stage II and stage III with IV were not statistically significant (*P* = 0.129, *P* = 0.286; respectively). Together with radical resection, these 2 systems were both significant in univariate and multivariate analysis (*P* < 0.05).

The newly updated WHO 2010 grading classifications and the AJCC 2010 staging systems could consistently reflect the clinical outcome of patients with surgically resected p-NETs. Meanwhile, both criteria could be independent predictors for survival analysis of p-NETs.

## INTRODUCTION

Pancreatic neuroendocrine tumors (p-NETs), namely islet cell tumors, are a heterogeneous group of malignancies with a common practice to label them as functional if patients present the symptoms related to hormone over production, such as insulinoma, gastrinoma, glucagonoma, etc, and nonfunctional if they do not.^1^ With an incidence of less than 5 per 1,000,000 each year, p-NETs are still uncommon, accounting for approximately 1% to 2% of all pancreatic tumors,^2–4^ though they showed an increasing tendency in recent decades.^5^

Owing to the rarity and heterogeneity, the ability to stratify patients with p-NETs into prognostic groups for survival analysis has been limited by the absence of a commonly accepted staging classification, even though they have evolved over 100 years since firstly reported.^6^ In 2000, based on the clinicopathologic features of neuroendocrine tumors, the World Health Organization (WHO) established for the first time a common scheme, which classified p-NETs into 3 categories.^7^ This classification was then updated and reclassified in 2010 into 4 main groups: neuroendocrine tumor G1 (NET G1), neuroendocrine tumor G2 (NET G2), neuroendocrine carcinoma G3 (NEC G3), and mixed adeno and neuroendocrine carcinoma (MANEC) to gain a widespread acceptance in clinical practice.^8^ In addition, also in 2010, the American Joint Committee on Cancer (AJCC) proposed an available tumor-node metastasis (TNM) staging system for p-NETs (ie, the seventh edition of AJCC staging manual), which was initially applied to the pancreatic exocrine adenocarcinoma.^9^ This new AJCC manual divided p-NETs into 4 stages, which distinguished between localized tumors (stage I), locally advanced but resectable tumors (stage II), locally advanced and unresectable tumors (stage III), and distantly metastasized tumors (stage IV).

The newly updated WHO 2010 grading classifications and the AJCC 2010 TNM staging systems differ greatly from each other, because the former one makes an important step toward defining the diverse tumor biology of p-NETs, whereas the latter reflects the time of diagnosis, rather than the tumor's inherent malignant potential. Therefore, presence of these 2 systems for p-NETs might raise clinical concerns of potential confusions in patient management. Meanwhile, in view of the more indolent and less malignant biologic behaviors, p-NETs have been considered to own better long-term survivals than pancreatic exocrine tumors, associated with a relatively higher rate of resection.^10,11^ So, use of a common staging system for 2 different disease processes, although convenient, might be oversimplified or inapplicable.

The clinical and prognostic value of the AJCC seventh TNM staging manual for p-NETs, yet no consensus, has already been validated in scanty mono-institutional series.^12–15^ On the contrary, however, appraisal of the newly updated WHO 2010 grading classifications has not been rigorously accomplished so far. To the best of our knowledge, the current analysis represented the first attempt to validate the new WHO grading systems. Therefore, based on the data of the eligible patients in our single institution, we aimed to analyze the clinical characteristics of p-NETs by applying the new WHO criteria in a high-volume surgical unit; to evaluate the clinical consistency of the new WHO 2010 grading classifications and the AJCC seventh staging manual on survivals for patients with surgically resected p-NETs; and to assess the prognostic value of both systems for survival analyses of p-NETs.

## MATERIALS AND METHODS

### Patient Selection^16^

This research was approved by the local ethics committee, and written consent was provided for patient information to be used for research purposes. Data of 120 consecutive patients from January 2004 to February 2014 in surgical departments of West China Hospital of Sichuan University, including patients’ demographics (sex and age), clinical presentations at admission (functional status), pathologic analyses, surgical procedures, and in-hospital stays, etc, were retrospectively collected from their electronic and/or article-based medical records. All patients were surgically treated and histologically diagnosed as p-NETs, whereas those with only clinical suspicion but not postoperatively pathologic confirmations of p-NETs were not enrolled in this study. All neoplasms originated from pancreas, whereas patients with tumors arising from the Vater ampulla, bile duct, duodenum, or retroperitoneal space were excluded. All tumors were sporadic, and patients with hereditary syndrome, such as multiple endocrine neoplasia type I, von Hippel-Lindau syndrome, and neurofibromatosis were not included as well. We also excluded in our research few patients who received postoperatively pharmaceutical treatments.

### Tumor Characteristics^17^

Diagnosis of p-NETs was totally pathologically confirmed based on histologic analysis and immunohistochemical staining of surgical specimens or biopsy samples. Features of tumor (size, location, lymph invasion, distant metastasis, surgical margin, component, mitotic count, Ki-67 positive rate, etc.) were mainly referred to the intraoperative findings by surgeons and ultimate pathologic analyses by pathologists of our hospital. The newly updated WHO 2010 grading classifications were quoted as follows: NET G1 (mitotic count: <2/10 high power fields HPF, Ki-67 <2%); NET G2 (mitotic count: 2–20/10 HPF, Ki-67: 3%–20%); NEC G3 mitotic count: >20/10 HPF, Ki-67 >20%); MANEC (mixed adeno-neuroendocrine carcinoma: 30% of either component required). Both this new WHO criteria and the AJCC seventh TNM staging manual were applied to assess the clinical outcome of each patient with surgically resected p-NETs.

### Follow-Up and Survival

We conducted telephone, office visit, and outpatient clinic to follow-up these eligible patients from August to October 2014, giving a potential follow-up time from 5.93 months to 125.67 months. Patients who were lost to follow-up were not enrolled in this study. Overall survival (OS) was defined as the number of months from the date of resection to the time of death or last contact. Deaths classified as not being related to the disease of p-NETs were also excluded when selecting patients.

### Statistical Analyses

Data were presented as mean ± standard error of mean or median for quantitative variables, or as numbers and their frequencies as proportions (%) for categorical variables unless otherwise indicated, which were then compared by Student *t* tests, analysis of variance or χ^2^ tests according to variable distribution wherever possible. We performed analyses of survival with Kaplan-Meier curves and comparisons among factors using log-rank test. Univariate and multivariate analyses were finally applied to assess the prognostic value of the new WHO 2010 grading classifications and the AJCC seventh staging manual for p-NETs by Cox regression proportional hazards model. Statistics was considered significant when P value of two sides was below 0.05. Statistical analyses were performed by IBM SPSS 17.0 statistical software.

## RESULTS

### Patient Characteristics

A total of 120 eligible and consecutive patients who were surgically treated and histologically diagnosed as p-NETs from January 2004 to February 2014 in our single institution were enrolled in our study, whose clinical-pathologic data regarding demographics, tumor characteristics, gradings, stages, etc, were summarized in detail in Table 1. Follow-up was began in August and finished in October, 2014, eventually developing a median follow-up time of 47.51 months and a mean of 50.72 ± 31.89 months (range: 5.93–125.67 months), with 34 patients (28.3%) followed to death.

**TABLE 1:**
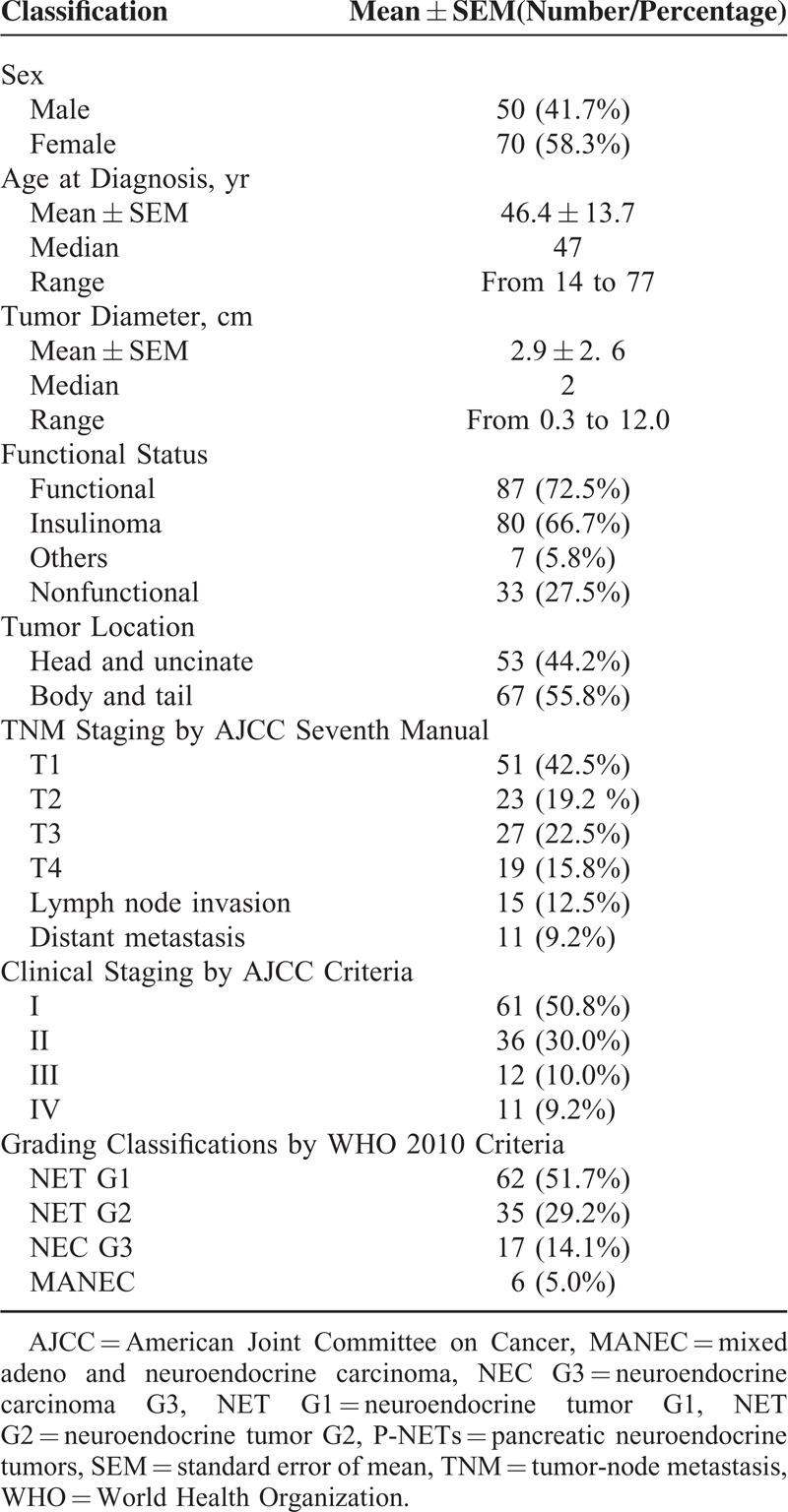
Clinical Features of Patients With Pancreatic Neuroendocrine Tumors in our Cohort Research

### Descriptions by World Health Organization Grading Systems

As was shown in Table 2, all selected patients were assigned through the newly updated WHO 2010 grading classifications, with a distribution of 62, 35, 17, and 6 patients for NET G1, NET G2, NEC G3, and MANEC, respectively. The mean age at initial diagnosis of all patients was 46.4 ± 13.7 years, presenting no significant difference among all grading groups (*P* = 0.978). Tumors of NET G1 and NET G2 seemed more frequently occurring in the body and tail of pancreas (36/62, 23/35; respectively), whereas those of NEC G3 and MANEC in pancreatic head and uncinate (11/17, 4/6; respectively). Statistically significant difference could be seen in tumor dimension (*P* < 0.001). Diameter of NET G1 was notably smaller than that of NET G2, NEC G3, and MANEC (*P* < 0.001, *P* < 0.001, *P* < 0.001; respectively), as well as comparison of NET G2 with NEC G3 and MANEC (*P* < 0.001, *P* = 0.016; respectively); whereas no statistical difference was detected between NEC G3 and MANEC (*P* = 0.425). Tumors with NET G1 and NET G2 were mostly in stage I or II (62/62, 32/35; respectively), yet those with NEC G3 and MANEC were largely in stage III or IV (14/17, 6/6; respectively). Finally, 11.3% patients with NET G1 were followed to death, whereas 25.7%, 70.6%, and 100% patients were dead in the left 3 respective groups.

**TABLE 2:**
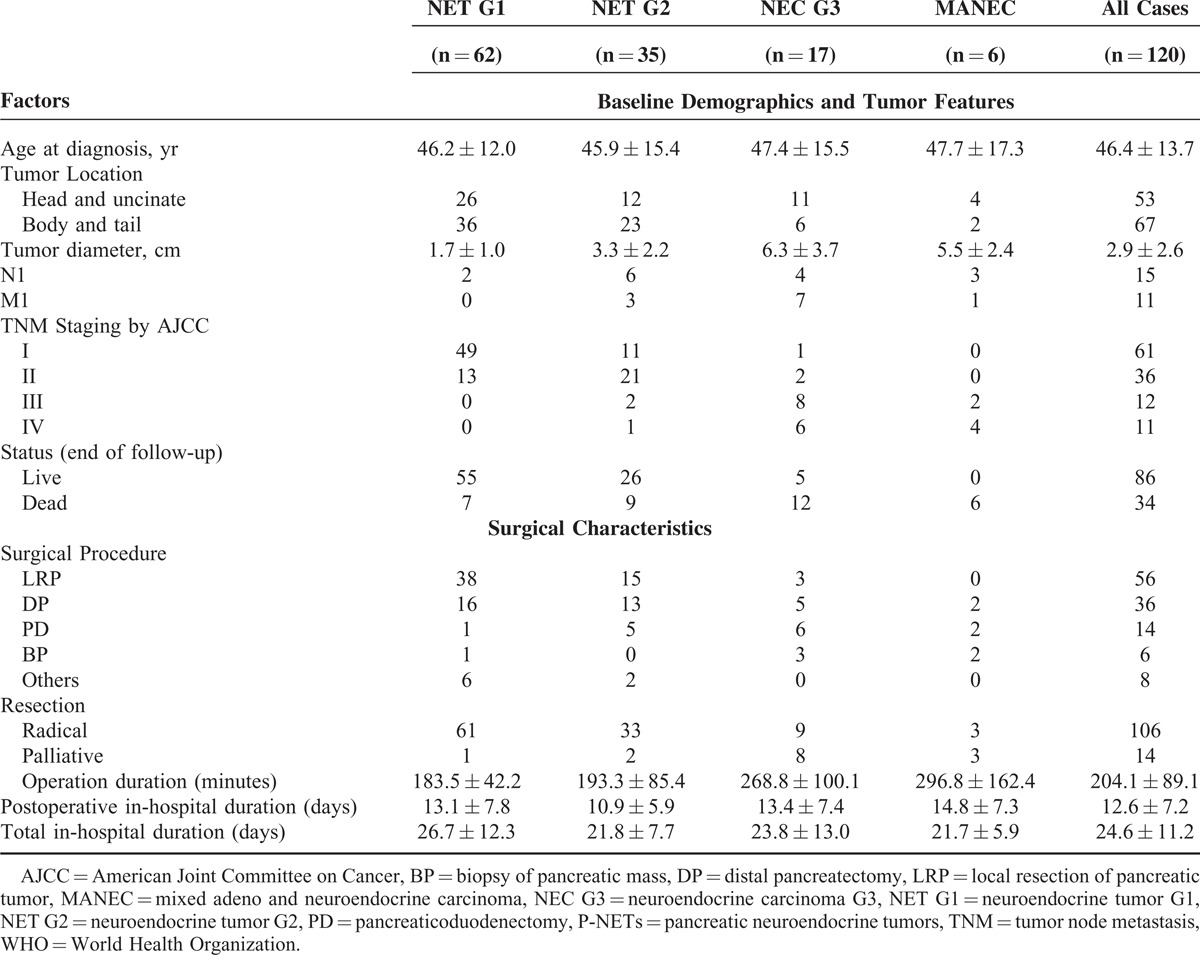
Distributions of Pancreatic Neuroendocrine Tumors With Different World Health Organization 2010 Grading Classification

All eligible patients underwent operational treatments, whose surgical features could also been found in detail in Table 2. Of all the 120 patients, 88.3% patients experienced radical resection, in which the most common performed procedure were local resection of pancreatic tumor (LRP, 46.7%), followed by distal pancreatectomy (DP, 30%),and pancreaticoduodenectomy (11.7%). Palliative operations were conducted on 14 patients, including biopsy of pancreatic mass (BP) for the unresectable tumors and reconstruction surgery of digestive tract for the obstruction symptoms of gastrointestinal tract and bile duct. In terms of the new WHO grading classifications, all patients but 1 with NET G1 and all but 2 with NET G2 underwent radical resections, in which LRP and DP were the most common procedures (87.1%, 80%; respectively). For tumors with NEC G3 and MANEC, palliative resections were performed on 8 and 3 patients (47.1%, 50%; respectively). The mean and median operation duration of total group was 204.1 ± 89.1 minutes and 180.0 minutes, respectively (range: 100–366 minutes), associated with an obvious difference among grading groups (*P* < 0.001). Particularly, operating time with NET G1 was statistically shorter than that with NEC G3 and MANEC (*P* < 0.001, *P* = 0.002; respectively), as well as that with NET G2 compared with NEC G3 and MANEC (*P* = 0.002, *P* = 0.005; respectively); whereas that between NET G1 and NET G2, NEC G3, and MANEC did not present any statistically significant difference (*P* = 0.575, *P* = 0.479; respectively). The overall mean postoperative and total in-hospital duration was respectively 12.6 ± 7.2 days and 24.6 ± 11.2 days, in which no notable difference was computed among the entirety (*P* = 0.416, *P* = 0.185; respectively), except the total in-hospital stay between NET G1 and NET G2 (*P* = 0.040).

### Survivals by American Joint Committee on Cancer and World Health Organization Criteria^16^

A TNM stage was expectedly assigned for each patient according to the new AJCC 2010 staging manual, which was also described in details in Table 1. There were respectively 51, 23, 27, and 19 patients from T1 to T4 by this criterion. Fifteen patients were pathologically confirmed to have lymph node invasion, whereas 11 patients present distant metastases. As far as the clinical staging was concerned, stage I, II, III, and IV by the AJCC seventh staging systems were defined in 61, 36, 12, and 11 patients, respectively.

**FIGURE 1:**
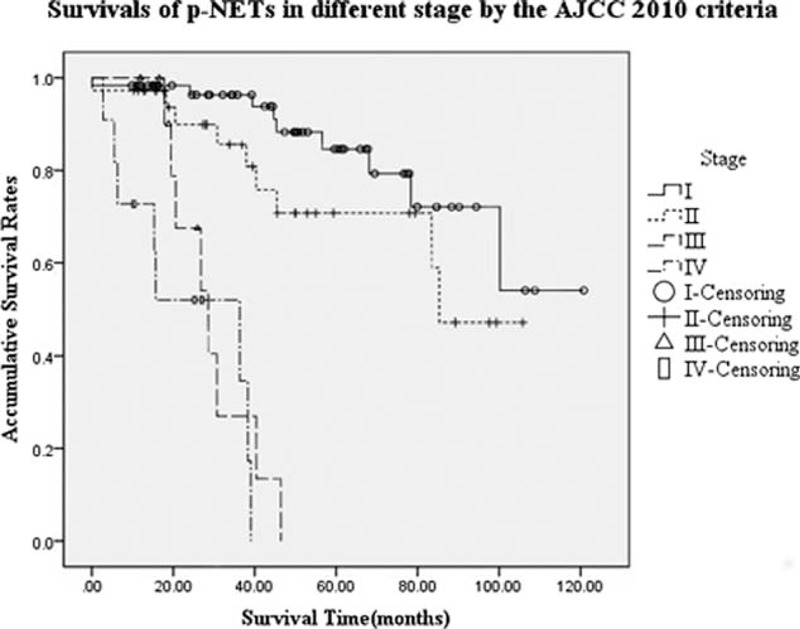
Survivals of pancreatic neuroendocrine tumors in different stage by the AJCC seventh staging manual. Differences of survivals of stage I with stage III and IV were significant (*P* < 0.001, *P* < 0.001; respectively), as well as those of stage II with III and IV (*P* < 0.001, *P* < 0.001; respectively); whereas comparisons of stage I with stage II and stage III with IV were not significant (*P* = 0.129, *P* = 0.286; respectively).

The 5-and 3-year OS rates for AJCC criteria stage I to IV were 84.6%, 70.7%, NA (could not be calculated), NA and 96.3%, 85.6%, 27.0%, 34.6%, respectively (*P* < 0.001, Fig. 1). Median survival time (MST) for each stage was NA, 85.3, 28.6, and 36.3 months, respectively. Differences of survival of stage I with stage III and IV were statistically significant (*P* < 0.001, *P* < 0.001; respectively). Similar results occurred again when comparing stage II with III and IV (*P* < 0.001, *P* < 0.001; respectively); whereas comparisons of stage I with stage II and stage III with IV did not present any notable difference (*P* = 0.129, *P* = 0.286; respectively).

**FIGURE 2:**
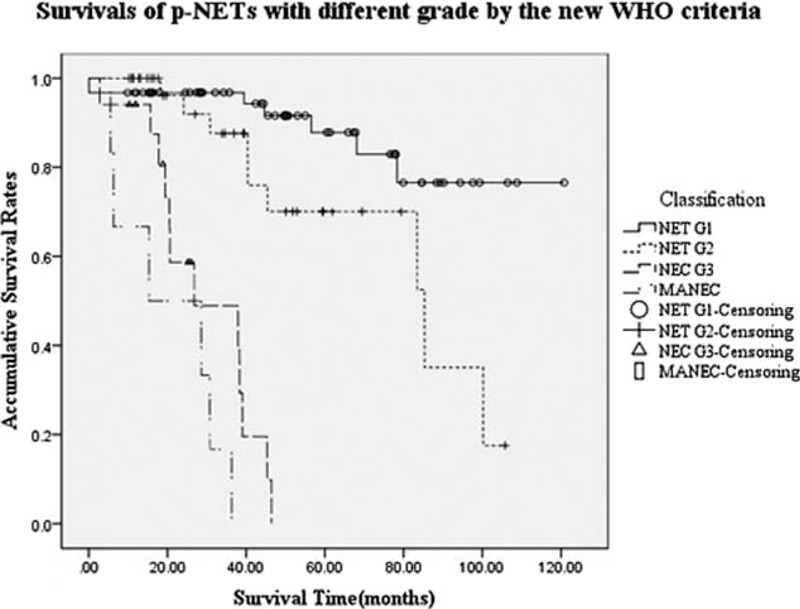
Survivals of pancreatic neuroendocrine tumors with different grade by the new World Health Organization 2010 grading classifications. Patients with NET G1 or NET G2 got a better survival compared with those with NEC G3 or mixed adeno and neuroendocrine carcinoma (*P* < 0.001). Survivals of NET G1 was longer than those of NET G2 (*P* = 0.023), whereas difference of survivals between NEC G3 and mixed adeno and neuroendocrine carcinoma present no obvious significance (*P* = 0.071). NET, neuroendocrine tumor.

In terms of the new WHO criteria, which, as we described before, were accordingly applied to all subjects with a distribution of 62, 35, 17, and 6 patients for each group, OS rates at 5 and 3 years were 87.8%, 70.1%, NA, NA and 96.8%, 87.6%, 48.9%, 16.7%, respectively (*P* < 0.001, Fig. 2). The MST for group NET G1, NET G2, NEC G3, and MANEC was NA, 85.3, 26.8, and 15.3 months, respectively. Patients with NET G1 or NET G2 obtained a statistically better survival compared with those with NEC G3 or MANEC (*P* < 0.001). In addition, survival of NET G1 was also statistically longer than that of NET G2 (*P* = 0.023), whereas difference of survival between NEC G3 and MANEC present no obvious significance (*P* = 0.071).

### Analyses for Prognostic Factors

In univariate analysis (Table 3), survivals of patients with surgically resected p-NETs were statistically associated with sex (male versus female), tumor dimension (over versus less than 2 cm), functional status (functional versus nonfunctional), radical resection (yes versus not), as well as the stages by AJCC seventh staging manual (stage I and II versus stage III and IV) and the gradings by WHO 2010 criterion (NET G1 and NET G2 versus NEC G3 and MANEC) (*P* < 0.05), whereas age (elder versus younger than 46 yrs), tumor location (head and uncinate versus body and tail), surgical procedure (LRP versus DP and pancreaticoduodenectomy) were not statistically significant (*P* > 0.05). The Cox multivariate regression proportional hazards model was subsequently performed to evaluate the prognostic value of these significant factors in univariate analysis (Table 4). We observed that only the AJCC seventh staging manual, the WHO 2010 grading classifications and radical resection were independent predictors for patients with surgically resected p-NETs.

**TABLE 3:**
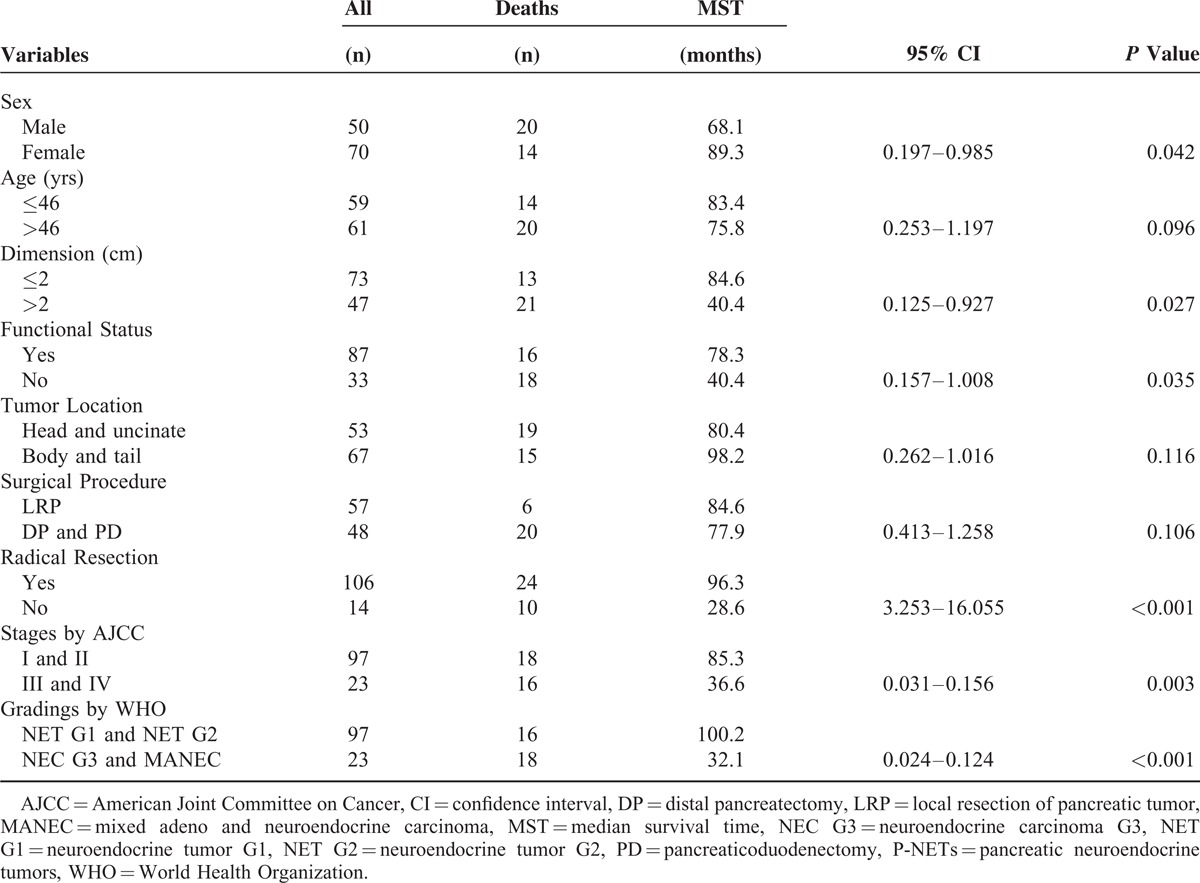
Univariate Analysis for Surgically Resected Pancreatic Neuroendocrine Tumors in our Institution (N = 120)

**TABLE 4:**
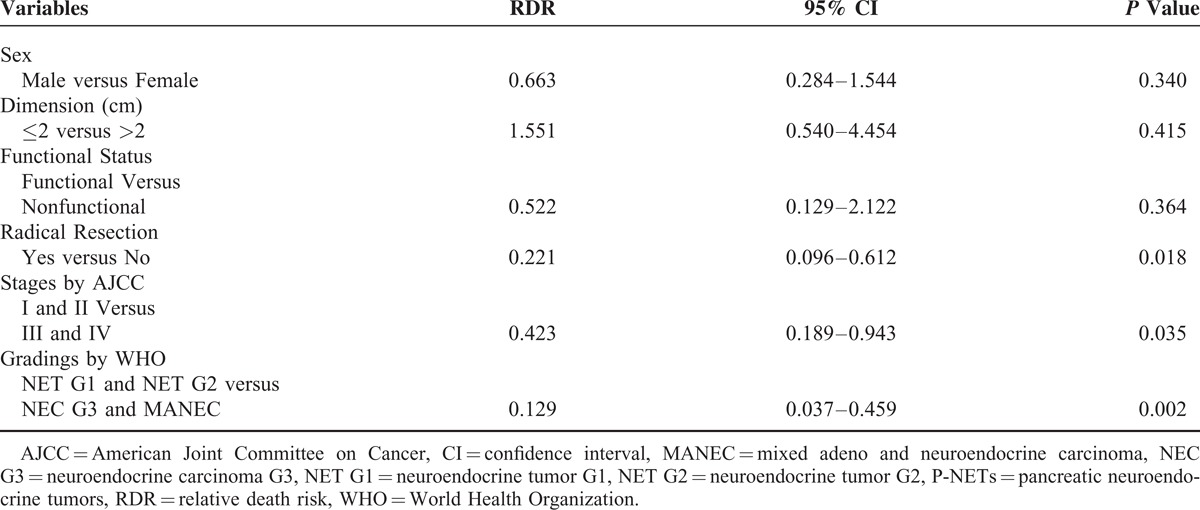
Multivariate Analysis of Potential Predictors for Surgically Resected Pancreatic Neuroendocrine Tumors

## DISCUSSION

In the current study, based on the data of the eligible patients in our single institution, we for the first time analyzed the clinical characteristics of p-NETs using the newly undated WHO 2010 grading classifications. We also demonstrated the clinical consistency of this WHO criterion and the AJCC seventh staging manual on survivals for patients with surgically resected p-NETs, which both successfully classified patients into 4 groups or stages with significant differences. Meanwhile, we firstly tried and finally validated the prognostic value of the new WHO grading systems for p-NETs, accompanied with the AJCC 2010 staging systems and radical resection.

As we mentioned before, because of the overall rare and heterogeneous behaviors with indolent malignancy, p-NETs have not been well studied as pancreatic adenocarcinoma. The absence of a uniform staging classification for p-NETs has hindered the ability to accurately predict their survivals.^18^ Also, the history of classifying p-NETs into groups for survival analysis has experienced a long and complicated evolution. In 1963, Williams and Sandler firstly classified carcinoid neoplasms based on the perceived embryological origin of these tumors in gastrointestinal tract.^19^ It, however, was not until 1995 that Capella et al^20^ particularly classified p-NETs by referring to angiolymphatic invasion, mitotic rate, tumor differentiation, as well as tumor size, distant metastases, presence of the hormonal syndrome. Then, on the basis of previous researches, the WHO officially introduced a whole grading system and classified p-NETs into 3 main types, which distinguished between well-differentiated endocrine tumor, well-differentiated endocrine carcinoma, and poorly differentiated endocrine carcinoma, also referring to the aforementioned clinicopathologic features of neuroendocrine tumors.^7^ Though the clinical and prognostic value had been largely validated by several studies,^21–25^ this classification was not widely accepted for its limited ability to predict the biologic aggressiveness of p-NETs, which ultimately led to the development of its update (ie, the WHO 2010 grading classifications).^8^ No practical evidence, however, has accumulated to assess the clinical value of the updated criteria, as well as its prognostic significance for p-NETs.

Moreover, since 1977 as we know, the staging guidelines for common solid organ tumors have been studied and developed by AJCC, which had not introduced a suitable and available TNM staging system for p-NETs until the year of 2010 (ie, the AJCC seventh staging manual).^9^ Nevertheless, this system was initially applied to the pancreatic exocrine adenocarcinoma, which similarly classified p-NETs into 4 stages. Though not the best,^26,27^ this new AJCC staging manual could be used for the survival analysis of p-NETs, whose clinical value has just recently been validated in numbered studies.^12–15^

In agreement with previous studies,^12–14^ the current study indicated that patients could also be successfully classified into 4 stages by the AJCC seventh staging manual. Consistently, patients in stage I showed a better survival than those in stage III and IV (*P* < 0.001, *P* < 0.001; respectively), as well as the comparisons of stage II with III and IV (*P* < 0.001, *P* < 0.001; respectively), whereas differences of stage I with stage II and stage III with IV were not statistically significant (*P* = 0.129, *P* = 0.286; respectively). Interestingly, we observed that in the current study, with a MST of 28.6 and 36.3 months respectively, patients in stage IV seemed to obtain a longer survival time than those in stage III, though this trend did not achieve any statistical significance (*P* = 0.286). This might be correlated with the special definitions by the AJCC seventh staging systems on stage III (ie, locally advanced and unresectable tumors) and stage IV (ie, distantly metastasized tumors). Because it has been widely accepted that surgery is the only potentially curative treatment of p-NETs if radical resection (R0 resection), including the primary tumor and even metastasis was achieved.^5,28^

On the contrary, as we described before, the new WHO criteria were also expectably assigned to 4 groups with different survival (*P* < 0.001). Similarly, patients with NET G1 or NET G2 obtained a statistically longer survival time compared with those with NEC G3 or MANEC (*P* < 0.001). Though survival time of NET G1 was statistically longer than that of NET G2 (*P* = 0.023), difference of survival between NEC G3 and MANEC present no obvious significance as well (*P* = 0.071). This result made it clear that the survival analyses of our data of our single institution have demonstrated the clinical consistency of the new WHO 2010 grading classifications and the AJCC seventh staging systems for the survival of p-NETs.

Many factors associated with predicting the survivals of p-NETs have been validated.^5,29–32^ In our research (Table 3), patient age, tumor location, and surgical procedures were not statistically correlated with the survival of p-NETs (*P* = 0.096, *P* = 0.116, *P* = 0.106; respectively), whereas the rest of 6 variables were all statistically significant (*P* < 0.05). Further analyses by Cox multivariate regression proportional hazards model confirmed the AJCC seventh staging manual as an independent predictor for the prognosis of p-NETs, as preciously validated by scanty reported studies.^12,13,27^ Our analyses also indicated the WHO 2010 grading classifications was another independent factor for predicting the survival of patients with surgically resected p-NETs, which meant the first successful attempt to validate the prognostic value of this new WHO criterion.

The major limitation of our study is its nature for the potential error and variation in collecting relevant data. What's more, we just studied the data of p-NETs in our single center by using these 2 criteria of the newly updated WHO 2010 grading classifications and the AJCC seventh staging manual in 2010. Actually, another TNM staging systems specially for p-NETs introduced in 2006 by the European Neuroendocrine Tumor Society (ENETS) have been also widely used in clinic.^33^ Moreover, the ENETS 2006 TNM staging systems have been validated to be superior to the AJCC 2010 staging manual, though both systems were independent predictors for the survival analyses of p-NETs.^27^ Thirdly, our colleagues have simultaneously evaluated the clinical consistency of the new WHO 2010 grading and the ENETS 2006 TNM staging systems on the surgical outcome for patients with p-NETs, who also validated the prognostic value of both criteria with different emphasis.^34^ Therefore, some in-depth evaluations or comparisons of different classification by WHO, AJCC, and ENETS are still needed to be further researched.

## CONCLUSIONS

In a word, we analyzed the clinical features of p-NETs in the current study by using the newly undated WHO 2010 grading classifications and the AJCC seventh staging manual in 2010. We meanwhile demonstrated that these 2 criteria could consistently reflect the clinical outcome of patients with surgically resected p-NETs. Subsequently, besides surgical margin, the new WHO grading classifications and the AJCC seventh staging manual were also validated to own each predicting value for the survival of patients with surgically resected p-NETs.
