# Survival Analyses for Patients With Surgically Resected Pancreatic Neuroendocrine Tumors by World Health Organization 2010 Grading Classifications and American Joint Committee on Cancer 2010 Staging Systems: Erratum

**DOI:** 10.1097/01.md.0000480962.17450.7d

**Published:** 2016-02-12

In the article “Survival Analyses for Patients With Surgically Resected Pancreatic Neuroendocrine Tumors by World Health Organization 2010 Grading Classifications and American Joint Committee on Cancer 2010 Staging Systems”,^1^ which appeared in Volume 94, Issue 48 of *Medicine*, the names of Dr. Zeng and Dr. Zhang were incorrectly reversed in the byline. Dr. Zhang should have been credited as an equal contributor to the manuscript. Also, Dr. Panagis Lykoudis should have been credited as the editor of the manuscript. The article has since been corrected online.
